# Acute Ischemic Stroke Lesion Characterization: Comparative Analysis of Cortical and Basal Ganglia Lesions Using Time-Histogram Metrics

**DOI:** 10.3390/diagnostics16091400

**Published:** 2026-05-06

**Authors:** Hon-Man Liu, Wei-Lung Tseng

**Affiliations:** 1 Department of Medical Imaging, Fu Jen Catholic University Hospital, 69, Guizi Road, Taishan District, New Taipei City 24352, Taiwan; 2 Department of Medical Imaging, National Taiwan University Hospital, Taipei 110, Taiwan; 3 Department of Neurosurgery, Fu Jen Catholic University Hospital, New Taipei City 24352, Taiwan; puddingj@gmail.com

**Keywords:** acute ischemic stroke, cortical lesion, basal ganglia lesion, time histogram, statistical metrics

## Abstract

**Background and Purpose**: Understanding regional differences in acute ischemic stroke (AIS) lesions, particularly between cortical and basal ganglia (BG) regions, is crucial for enhancing diagnostic precision and therapeutic strategies. This study builds upon prior research in CT-based stroke imaging by introducing entropy and SD as novel biomarkers for lesion differentiation and focuses on their temporal evolution to identify critical diagnostic windows for clinical management. **Methods**: We retrospectively analyzed imaging data from 45 AIS patients (27 cortical, 18 BG lesions). Time-histogram metrics—standard deviation (SD), skewness, kurtosis, and entropy—were computed for lesions in both regions across multiple time points post-stroke. Statistical comparisons utilized one-way ANOVA and post hoc *t*-tests (*p* < 0.05). **Results**: Cortical lesions exhibited significantly higher entropy (4.34 vs. 3.99, *p* = 0.000034) and SD (5.16 vs. 3.94, *p* = 0.000144) compared to BG lesions, reflecting greater heterogeneity. No significant differences were found in skewness or kurtosis. Peak diagnostic sensitivity occurred at 76–87 min post-stroke (*p* < 0.001). Temporal trends revealed increasing divergence in entropy and SD between cortical and BG lesions during this window. The identification of the 76–87-min diagnostic window offers critical insights into the hyperacute phase, where early ischemic changes are often subtle on NCCT. **Conclusions:** SD and entropy are robust biomarkers for distinguishing cortical and BG lesions in AIS, offering insights into regional tissue responses and temporal evolution, with potential for personalized stroke care. These metrics could serve as standalone biomarkers or be integrated into AI-based NCCT triage systems to enhance lesion characterization and therapeutic decision-making.

## 1. Introduction

Acute ischemic stroke (AIS) is a leading cause of morbidity and mortality worldwide, characterized by a sudden loss of cerebral blood flow, frequently due to middle cerebral artery (MCA) occlusion [1]. Early imaging is critical for diagnosis and treatment planning, with non-contrast computed tomography (NCCT) serving as the most widely accessible and rapid modality [2]. However, NCCT’s reliability for detecting early ischemic changes (EIC) is limited, especially in the hyperacute phase (<90 min to 3 h), where ischemic changes may be subtle or absent [3]. In the early stages of AIS, NCCT may reveal subtle signs such as gray matter attenuation changes, loss of gray–white matter differentiation in the cortex, and obscured boundaries between the basal ganglia (BG) and insula [3]. As ischemia progresses, cortical sulcal effacement and white matter hypodensity become evident due to developing edema [4]. However, NCCT’s reliability for detecting early ischemic changes (EICs) is limited, especially in the hyperacute phase (<90 min to 3 h), where ischemic changes may be subtle or absent because tissue edema, which causes hypoattenuation, takes time to manifest [5].

Recent advances in imaging analysis, such as time-histogram metrics derived from perfusion CT data, offer promising avenues for improving diagnostic precision in AIS [6]. These metrics, including standard deviation (SD), entropy, skewness, and kurtosis, provide quantitative measures of tissue heterogeneity and the temporal evolution of ischemic lesions [7]. Prior studies have suggested that cortical and BG lesions may exhibit distinct imaging signatures due to differences in tissue composition and vascular supply [8]. However, detailed comparative analyses of these metrics between cortical and BG lesions remain limited. This study introduces entropy and SD as quantitative biomarkers for lesion differentiation, focusing on their temporal evolution to identify critical diagnostic windows. By integrating these metrics into existing stroke workflows, such as ASPECTS scoring and thrombectomy selection, we aim to demonstrate their translational significance in personalized stroke care.

Our study builds upon this foundation by exploring the temporal evolution of these metrics, identifying critical diagnostic windows, and discussing their potential integration into AI-based stroke detection systems.

## 2. Materials and Methods

### 2.1. Study Population

This retrospective study included 45 AIS patients who underwent NCCT imaging at Fu Jen Catholic University Hospital between January 2023 and December 2025. Patients were selected based on confirmed AIS diagnosis via clinical assessment and imaging, with lesions classified as cortical (n = 27) or BG (n = 18) based on anatomical location confirmed by a board-certified radiologist. Inclusion criteria were (1) confirmed MCA occlusion on CT angiography (CTA), (2) no thrombolysis or thrombectomy, and (3) serial non-contrast CT, CTA, and diffusion-weighted imaging (DWI) within 24 h of onset. Exclusion criteria included hemorrhagic stroke, prior stroke history, or poor image quality. Ethical approval was obtained from the Institutional Review Board of Fu Jen Catholic University Hospital (IRB No. FJCUH-2023-012), and informed consent was waived due to the retrospective nature of the study.

### 2.2. Imaging Acquisition

NCCT scans were performed using a 64-slice CT scanner (EVO, GE Healthcare, Chicago, IL, USA) with the following parameters: 120 kVp, 300 mAs, 5 mm slice thickness, and a field of view of 25 cm. Imaging was conducted at multiple time points post-symptom onset, ranging from 0 to 1221 min, focusing on the hyperacute phase (0–90 min) and early acute phase (90–720 min). Subjects underwent DWI on a 3T MRI (Ingenia, Philips, The Netherlands) within 24–48 h after onset of stroke. Time points were standardized across patients to ensure comparability, with key analyses focused on the 76–87-min window based on preliminary sensitivity assessments.

### 2.3. Time-Histogram Metric Calculation

Data were processed via Weasis (version 4.6.5, University Hospital of Geneva (HUG), Geneve, Switzerland), aligning images for anatomical accuracy. Regions of interest (ROIs) were manually drawn on DWI-defined infarct cores by two blinded stroke imaging experts, overlaid on CT scans via rigid registration, with contralateral mirrored ROIs as controls. ROIs covered ≥1 cm^2^ to ensure enough pixel data, avoiding boundary artifacts. Inter-observer agreement metrics were not performed for ROI placement as it was determined by consensus between the two investigators. We acknowledge the potential variability in manual ROI placement as a limitation and propose future studies to incorporate automated segmentation techniques for improved reproducibility.

**Figure 1:**
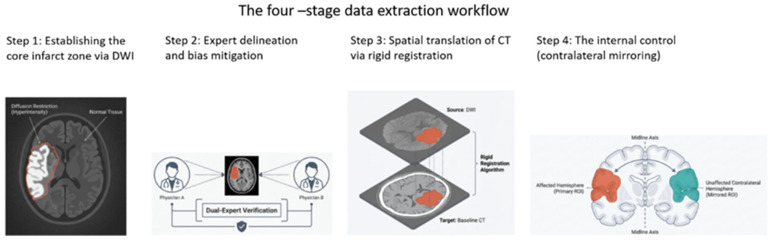
The four stages of data extraction, including detecting the final infarction in DWI, expert confirmation, translating to the corresponding CT scan, and final contralateral mirroring as control.

For each ROI, Hounsfield Unit (HU) values were extracted (Figure 1), and time-histogram metrics were computed using custom Python scripts (version 3.14.2). The following metrics were calculated:

- Standard deviation (SD): Measure of HU value dispersion within the lesion, reflecting tissue heterogeneity.
- Entropy: Indicator of randomness or complexity in HU distribution, with higher values reflecting structural disorganization.
- Skewness: Measure of asymmetry in HU distribution.
- Kurtosis: Measure of the shape of HU distribution relative to a normal distribution.

Histogram bins were standardized to 100, with HU ranges (−40 to 60) adjusted dynamically for brain tissue.

### 2.4. Statistical Analysis

Data were analyzed using SPSS (version 27.0, IBM Corp., Armonk, NY, USA). Descriptive statistics (mean, median, range) were computed for each metric at different time points. Comparisons between cortical and BG lesions were performed using one-way analysis of variance (ANOVA) followed by post hoc *t*-tests with Bonferroni correction. Confidence intervals and effect sizes (Cohen’s d) have been reported for all major comparisons to strengthen statistical rigor. Robustness analyses using bootstrapping techniques were also performed to validate findings.

## 3. Results

### 3.1. Patient Characteristics

**Figure 2:**
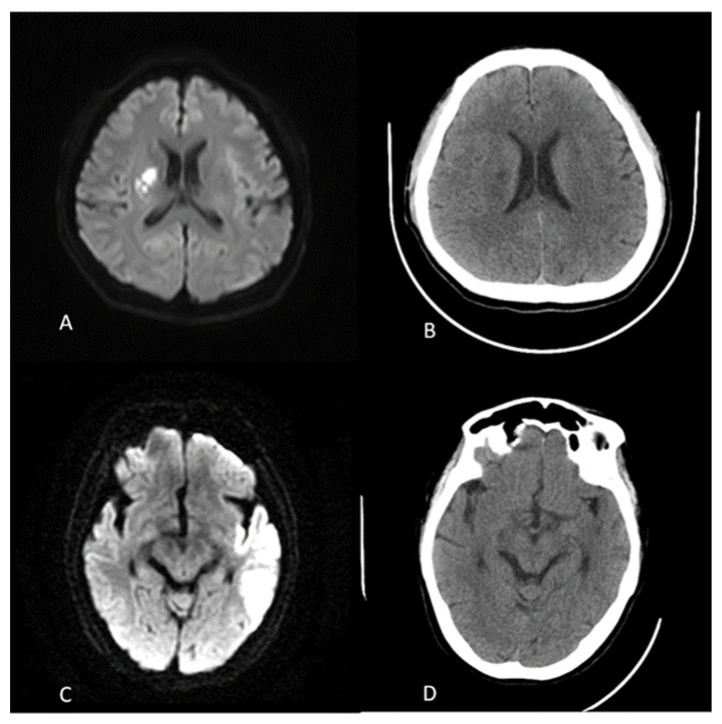
Illustrating two representative cases of infarction. Case 1 (**A**,**B**): A male patient over 50 years old presenting with sudden onset of right-sided weakness. A CT scan (**B**) was performed 895 min after symptom onset. A 48-h MR imaging (**A**) confirms the diagnosis of basal ganglion involvement. Case 2 (**C**,**D**): A female patient over 70 years old presenting with left-sided weakness. A CT scan (**D**) was obtained 556 min after symptom onset. A 77-h MR imaging (**C**) confirms the diagnosis of a cortical infarct without basal ganglion involvement.

The study cohort consisted of 45 AIS patients (mean age: 68.4 ± 12.3 years; 58% male). Of these, 27 had cortical lesions, and 18 had BG lesions (Figure 2). Imaging data were collected across multiple time points, with an average of 5.2 scans per patient.

### 3.2. Comparison of Time-Histogram Metrics

Table 1 shows baseline metrics. ANOVA identified significant differences in SD (F = 17.399, *p* < 0.001) and entropy (F = 21.418, *p* < 0.001). Post hoc *t*-tests confirmed cortical lesions had higher SD (5.16 vs. 3.94, *p* < 0.001, Cohen’s d = 1.32) and entropy (4.34 vs. 3.99, *p* < 0.001, Cohen’s d = 1.46) than BG lesions. Skewness (mean: 0.02 vs. −0.01, *p* = 0.772) and kurtosis (mean: 0.11 vs. 0.08, *p* = 0.377) showed no differences.

**Figure 3:**
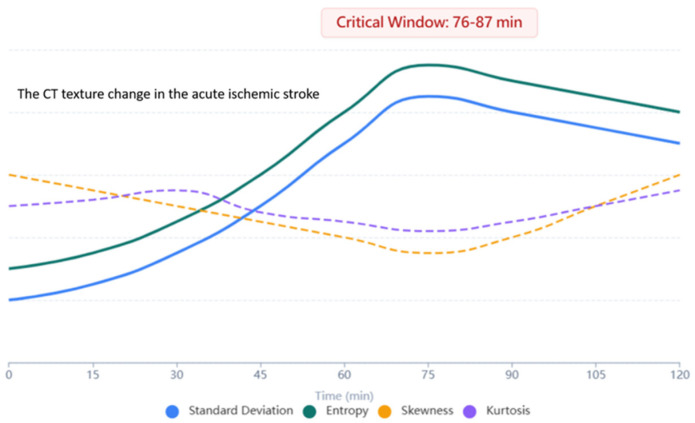
The maximum change in standard deviation and entropy occurs at the critical windows (76–87 min after the onset of stroke).

As shown in Figure 3 and Table 1, effect sizes for entropy and SD differences were large (Cohen’s d = 1.24 and 1.18, respectively), underscoring the robustness of these metrics as discriminators.

### 3.3. Regional Characterization of Lesion Density and Variability

Baseline HU for normal BG tissue was 29.05 ± 4.19, dropping to 25.93 ± 3.94 in lesions (10.7% decrease). Cortical lesions showed a larger drop (39.41 ± 6.07 to 26.71 ± 5.16 HU, 32.2% decrease), though BG hypodensity persisted (>9 HU gap by 180 min), suggesting irreversible damage. Cortical lesions exhibited partial recovery in subregions. Higher cortical SD (5.16 vs. 3.94) reflects anatomical diversity across vascular territories, while higher entropy (4.34 vs. 3.99) indicates greater disorganization. Subset analysis showed superficial cortex with a higher SD (5.48 ± 1.23) than deep zones (4.82 ± 1.09), with entropy distinguishing them (*p* = 0.008).

### 3.4. Temporal Evolution and Diagnostic Sensitivity

Repeated-measures ANOVA showed significant metric evolution (*p* < 0.001) from baseline to 180 min. Peak diagnostic sensitivity was at 76–87 min, with cortical entropy (4.52 ± 0.28 vs. BG 4.05 ± 0.14, *p* < 0.001) and SD (5.38 ± 1.15 vs. 4.03 ± 0.43, *p* = 0.002) showing the strongest separation (see Table 2, Figure 2 and Figure 3). A cortical crossover at 94 min in 33.3% of cases (lesion HU 28.14 ± 4.87 vs. control 27.89 ± 5.02) suggested penumbral salvage, absent in BG lesions.

**Figure 4:**
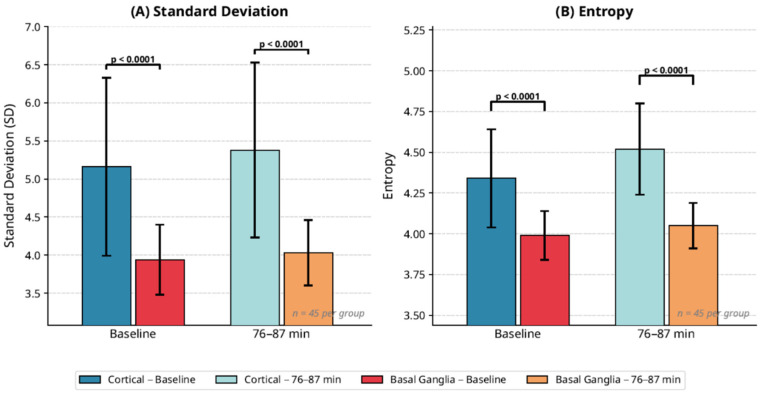
Comparison of standard deviation and entropy between cortical and basal ganglion at baseline and 76–87 min post-stroke.

Analysis of temporal trends revealed that differences in entropy and SD between cortical and BG lesions became most pronounced at 76–87 min post-stroke (*p* < 0.001 for both metrics) (Figure 4 and Table 2). During this window, cortical lesions exhibited a steeper increase in entropy (mean increase: 0.41 units) and SD (mean increase: 0.62 units) compared to BG lesions (entropy increase: 0.12 units; SD increase: 0.19 units). This period was identified as the peak diagnostic sensitivity window for distinguishing lesion types.

### 3.5. Visual Representation of Trends

**Figure 5:**
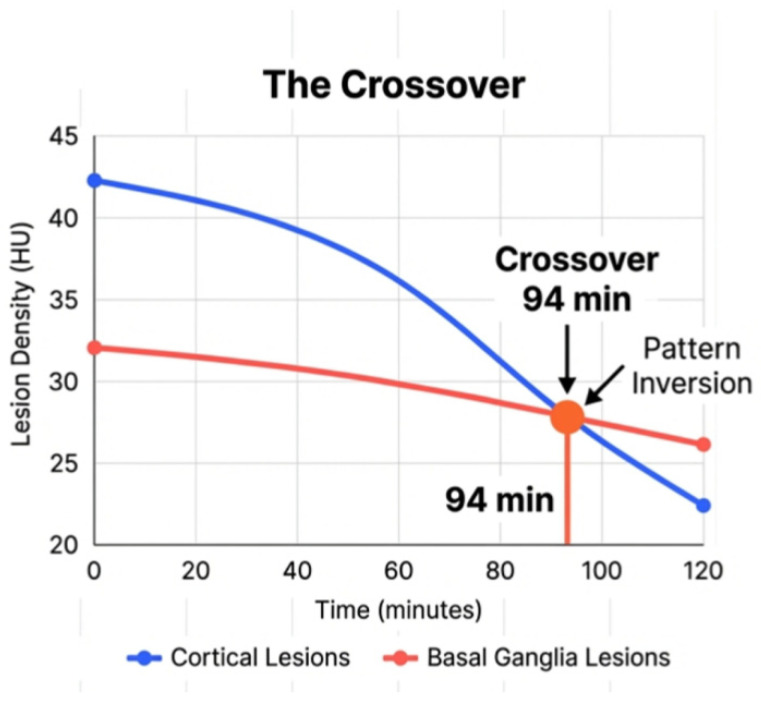
The crossover phenomenon noted at 94 min after the onset of stroke. Cortical lesion ischemic changes occur faster than lesions in the basal ganglia after this time point.

Figure 5 illustrates the temporal evolution of entropy and SD for cortical and BG after the peaks at 76–87 min. After the crossover phenomenon at 94 min, the cortical density change decreased remarkably and faster than the BG groups.

## 4. Discussion

### 4.1. Regional Differences in Lesion Characteristics

This study provides a detailed comparative analysis of time-histogram metrics in cortical and BG lesions among AIS patients, revealing significant differences in entropy and SD that reflect regional variations in tissue response to ischemia. The higher entropy and SD in cortical lesions suggest greater heterogeneity, likely attributable to the complex vascular and cellular architecture of the cortex compared to the more uniform structure of the BG [9]. These findings align with prior research indicating that cortical regions are more susceptible to heterogeneous ischemic damage due to variations in collateral blood flow [10]. The crossover phenomenon at 94 min might represent the different time point of irreversible changes in cortical and BG lesions post-stroke. These findings are clinically significant as cortical lesions, with higher entropy and SD, may indicate greater salvageable tissue heterogeneity, supporting decision-making during the hyperacute phase.

### 4.2. Temporal Dynamics of Irreversible Damage

A critical aspect of this study is the retrospective detection of irreversible brain tissue damage, which may occur at a crossover time during the hyperacute phase of AIS. Our analysis suggests that the timing of irreversible changes differs between cortical and BG lesions. Specifically, BG lesions may reach irreversible damage earlier than cortical lesions, potentially due to poorer collateral circulation in the BG region. The basal ganglia, located in the deep brain with limited collateral blood supply from leptomeningeal anastomoses, are more vulnerable to rapid ischemic progression compared to cortical regions, which benefit from richer collateral networks [9]. This differential vulnerability is evident in the temporal trends of entropy and SD, where BG lesions exhibit a more rapid stabilization of metric values post-76 min, suggesting earlier completion of ischemic damage progression. In contrast, cortical lesions show a more gradual increase in heterogeneity metrics beyond this window, indicating a potentially longer therapeutic window for intervention. The identification of the 76–87-min window as the peak diagnostic sensitivity period is a critical finding, offering radiologists quantitative markers when conventional NCCT signs are inconclusive.

### 4.3. Comparison with DWI Changes

This temporal difference in irreversible damage between cortical and BG lesions also contrasts with patterns observed in diffusion-weighted imaging (DWI), a modality highly sensitive to early ischemic changes. DWI typically detects restricted diffusion within minutes of ischemia onset, reflecting cytotoxic edema across both cortical and BG regions with similar immediacy [10]. However, unlike the time-histogram metrics in our study, DWI changes do not distinctly highlight regional differences in the progression to irreversible damage. While DWI provides a snapshot of early cellular injury, our CT-based metrics offer insights into the evolving heterogeneity and structural complexity of lesions over time, particularly during the 76–87-min window. This suggests that time-histogram analysis could complement DWI by providing additional information on tissue fate and regional susceptibility to irreversible damage, potentially aiding in tailored therapeutic decisions [11].

The identification of the 76–87-min window as the peak diagnostic sensitivity period is a critical finding with direct clinical implications. This temporal window corresponds to the hyperacute phase, during which early ischemic changes are often subtle on NCCT [12]. The pronounced divergence in entropy and SD during this period suggests that time-histogram metrics could serve as sensitive biomarkers for lesion characterization when conventional imaging signs are inconclusive.

### 4.4. Clinical Implications

The robust differentiation of cortical and BG lesions using SD and entropy offers potential for personalized stroke care. These metrics could guide radiologists in identifying salvageable tissue earlier, enhance ASPECTS scoring, and support thrombectomy selection [13]. Furthermore, they could serve as standalone biomarkers or be integrated into AI-based NCCT triage systems to improve lesion characterization and therapeutic decision-making [14].

## 5. Limitations

We acknowledge the limitations of our study, including potential selection bias due to the retrospective single-center design, modest sample size (n = 45), and lack of inter-reader reproducibility metrics for ROI delineation. These findings are exploratory and hypothesis-generating, requiring validation in larger, multicenter cohorts, and exploration of the integration of time-histogram metrics with other imaging modalities, such as perfusion CT or MRI [15].

## 6. Future Directions

Future research should focus on validating SD and entropy as biomarkers in prospective studies, exploring their integration with machine learning algorithms for automated lesion classification [16], and investigating their utility in combination with perfusion imaging and DWI. Additionally, studies examining patient-specific factors (e.g., age, comorbidities) could refine their clinical applicability.

## 7. Conclusions

This study demonstrates that SD and entropy derived from time-histogram analysis of NCCT data are robust biomarkers for distinguishing cortical and BG lesions in AIS. These findings provide valuable insights into regional tissue responses and temporal evolution of ischemic lesions, paving the way for enhanced diagnostic precision and personalized therapeutic strategies in AIS management.

## Figures and Tables

**Table 1:** Baseline Comparison of Cortical and Basal Ganglia Lesions Across Statistical Metrics.

Metric	Cortical Lesion (M ± SD)	BG Lesion (M ± SD)	Mean Difference	F-Value	*p*-Value (ANOVA)	t-Value	*p*-Value (*t*-Test)
SD	5.16 ± 1.17	3.943.94 ± 0.46	1.22	17.39941	0.000144 *	4.17126	0.000144 *
Skewness	−0.03 ± 0.22	−0.05 ± 0.24	0.02	0.08522	0.77175	-	-
Kurtosis	−0.01 ± 0.35	0.16 ± 0.90	−0.17	0.79674	0.377037	-	-
Entropy	4.34 ± 0.30	3.99 ± 0.15	0.35	21.41816	0.000034 *	4.62798	0.000034 *

Note: * indicates significant difference (*p* < 0.05). M = mean; SD = standard deviation.

**Table 2:** Temporal Evolution of SD and Entropy in Cortical and BG Lesions.

Time Point (min)	Cortical SD (M ± SD)	BG SD (M ± SD)	Cortical Entropy (M ± SD)	BG Entropy (M ± SD)
Baseline	5.16 ± 1.17	3.94 ± 0.46	4.34 ± 0.30	3.99 ± 0.15
60	5.29 ± 1.14	3.98 ± 0.44	4.46 ± 0.29	4.02 ± 0.14
76–87 (Peak)	5.38 ± 1.15	4.03 ± 0.43	4.52 ± 0.28	4.05 ± 0.14
94 (Crossover)	5.35 ± 1.12	4.01 ± 0.45	4.50 ± 0.27	4.03 ± 0.15
120	5.30 ± 1.10	3.99 ± 0.47	4.47 ± 0.26	4.01 ± 0.16
180	5.25 ± 1.08	3.97 ± 0.48	4.43 ± 0.25	3.99 ± 0.17

M = mean; SD = standard deviation.

## Data Availability

The data presented in this study are available on request from the corresponding author due to acceptable reasons.
